# Complete Response to Combined Chemotherapy and Anti-PD-1 Therapy for Recurrent Gallbladder Carcinosarcoma: A Case Report and Literature Review

**DOI:** 10.3389/fonc.2022.803454

**Published:** 2022-03-14

**Authors:** Qin-qin Liu, Hao-ming Lin, Hong-wei Han, Cai-ni Yang, Chao Liu, Rui Zhang

**Affiliations:** Guangdong Provincial Key Laboratory of Malignant Tumor Epigenetics and Gene Regulation and Department of Biliary-Pancreatic Surgery, Sun Yat-sen Memorial Hospital, Sun Yat-sen University, Guangzhou, China

**Keywords:** chemotherapy, anti-PD-1 therapy, combination therapy, complete response, recurrent gallbladder carcinosarcoma

## Abstract

**Background:**

Gallbladder carcinosarcoma (GBCS) is a rare and aggressive malignancy with extremely poor prognosis. Although surgery is regarded as the primary therapy for GBCS, the effective therapeutic strategies for unresected lesions have been poorly defined.

**Case Presentation:**

We presented a case of a 74-year-old male who underwent radical resection of gallbladder carcinoma at a local hospital. Seven months later, he was admitted to our hospital due to right upper abdominal discomfort. Postoperative radiological examinations showed multiple hepatic lesions, hilar lymph node metastasis, and main portal vein tumor thrombus. The pathological consultation results confirmed GBCS and immunohistochemical examinations revealed PD-L1 expression in 20% of tumor cells. Then, the patient received chemotherapy (Gemcitabine plus Oxaliplatin, GEMOX) in combination with anti-PD-1 therapy. After nine courses of the combination therapy, complete regression of the tumors was achieved with no evidence of relapse till now.

**Conclusions:**

We, for the first time, reported a patient with recurrent GBCS who benefited from the combined chemotherapy and immunotherapy, providing a potential effective management strategy for the refractory malignant tumor.

## Introduction

Carcinosarcoma is a rare malignant tumor consisting of carcinomatous and sarcomatous elements (1). The most commonly involved sites are uterus, ovaries, esophagus, thyroid, and larynx; however, cases of gallbladder carcinosarcoma (GBCS) are extremely rare with an incidence of less than 1% worldwide (2). Although surgical resection is recognized as the optimal treatment for GBCS, the prognosis remains unfavorable due to late diagnosis, early relapse, and aggressive biologic behaviors of the tumor. The gemcitabine-based chemotherapy regimens are commonly used for unresected gallbladder carcinoma, but the effects are uncertain with low response rates (3). Furthermore, the established therapeutic regimens are still unavailable because of limited understanding of the low-incident disease.

Recently, immunotherapy, a novel emerging approach for the regulation of anti-tumor immunity, has attracted extensive attention in tumor therapy. The immune checkpoint inhibitions (ICIs), targeting the programmed cell death 1 (PD-1)/programmed cell death ligand 1 (PD-L1) and cytotoxic T-lymphocyte antigen 4 (CTLA4), have shown potential therapeutic benefits in several solid tumors. Atezolizumab in combination with bevacizumab has been approved as first-line treatment for advanced hepatocellular carcinoma (4). The first-line treatment with Pembrolizumab has been proved to be efficacious for the management of advanced non—small-cell lung cancer with high PD-L1 expression (5). Despite these encouraging clinical outcomes, the role of immunotherapy in advanced biliary tract cancer (BTC) is still under exploration (6). We herein reported a rare case of a patient with recurrent GBCS who achieved complete tumor regression following the combined chemotherapy (Gemcitabine plus Oxaliplatin, GEMOX) and immunotherapy (Sintilimab).

## Case Presentation

**Figure 1:**
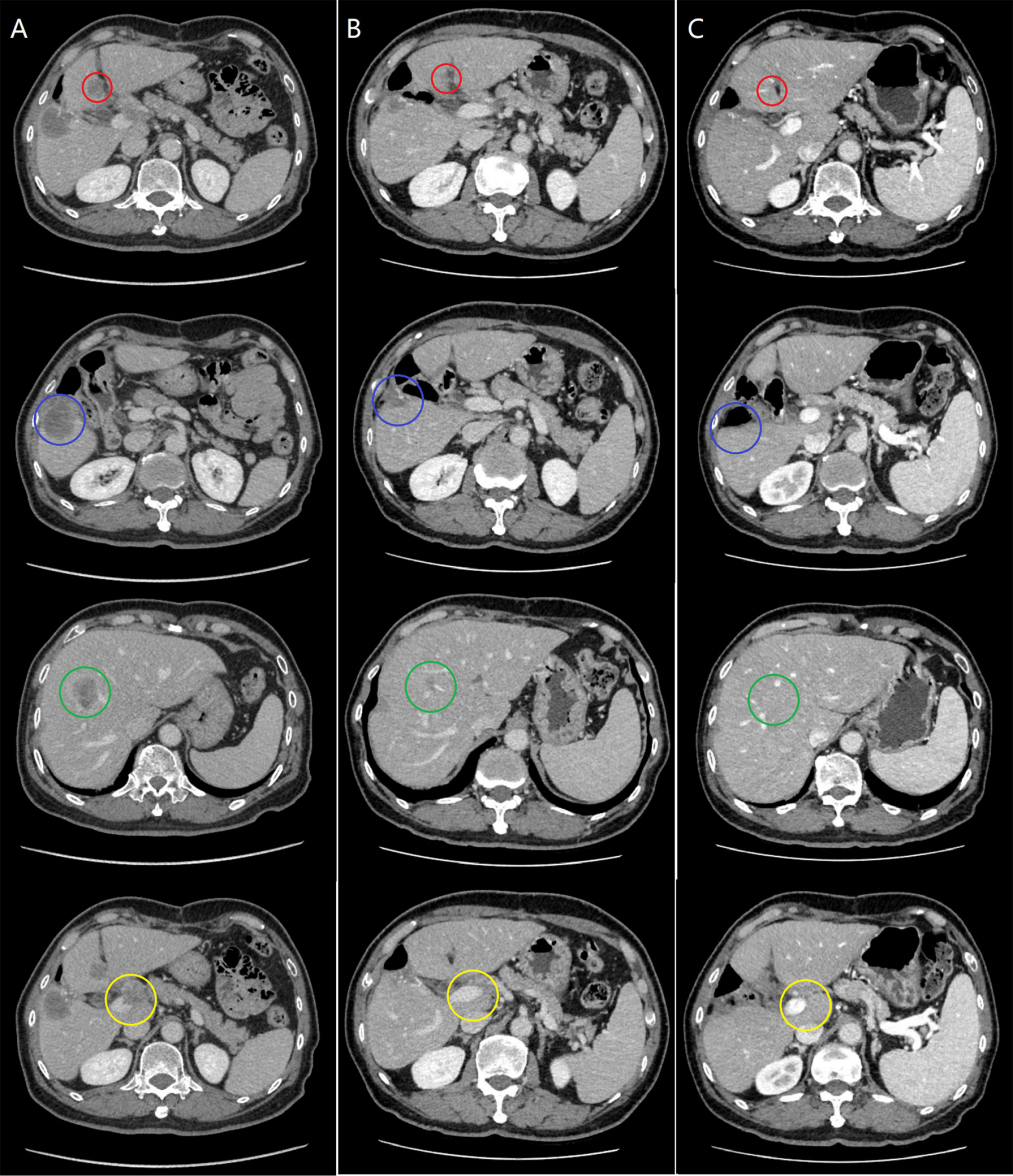
The evaluation of tumor response during the clinical course. **(A)** Pretreatment computed tomography (CT) revealed recurrent lesions in the segment IV (red circle), V (blue circle) and IV/VIII (green circle) of the liver, and a metastatic lesion in the hepatic hilum with tumor thrombus in the main portal vein (yellow circle). **(B)** After two courses of combination therapy, CT showed the intrahepatic lesions decreased in size, and both the lesion in the hepatic hilum and the main portal vein tumor thrombus disappeared. **(C)** After six courses of combination therapy, CT showed complete resolution of all the lesions.

**Figure 2:**
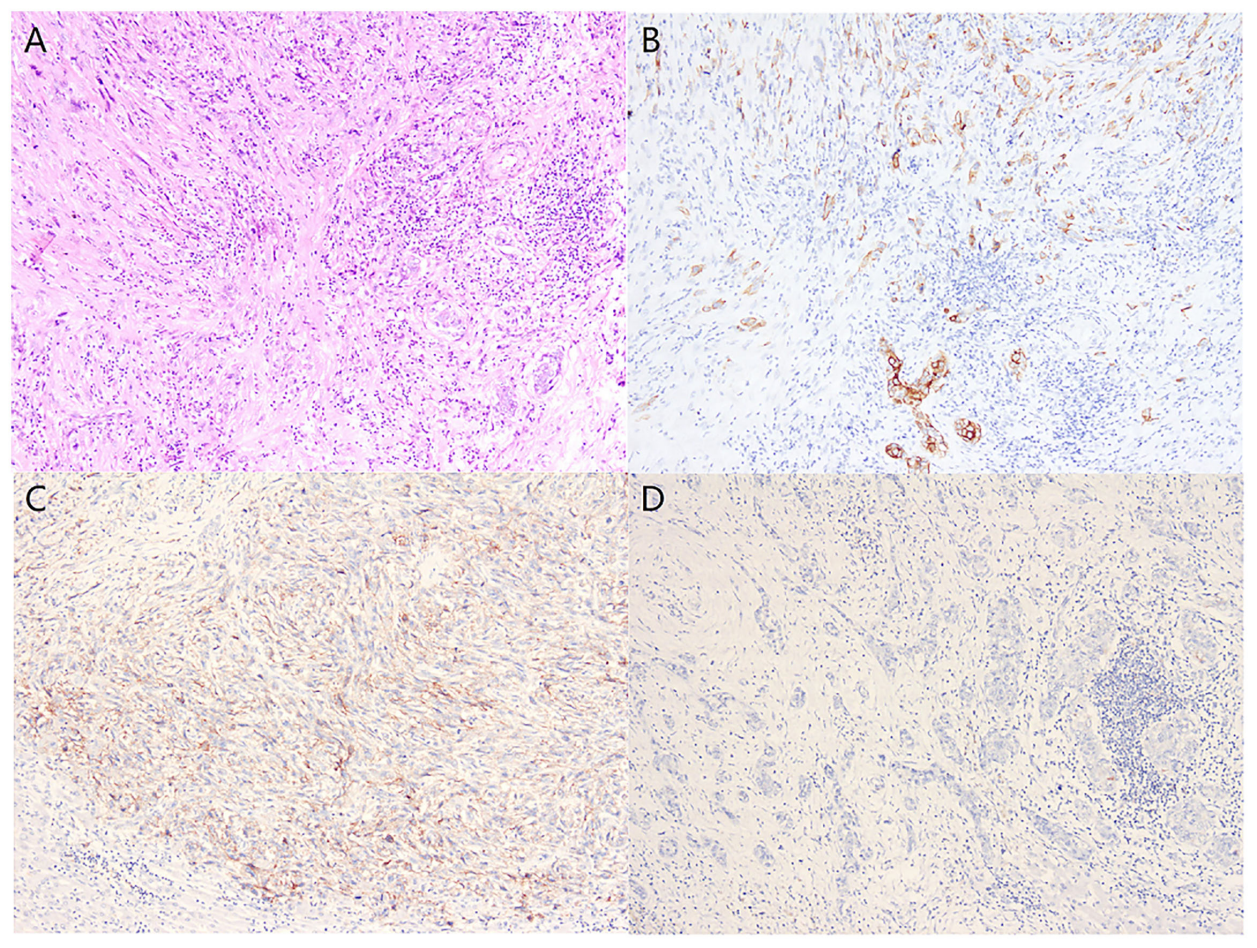
Histologically, the tumor tissue was composed of poorly differentiated adenocarcinoma and spindle cell sarcoma **(A)** (Hematoxylin and eosin stain, x100). Immunohistochemical examination revealed positive staining for CK in the carcinomatous and sarcomatous components **(B)**, PD-L1-positive expression in the sarcomatous components **(C)**, and PD-L1-negative expression in the carcinomatous components **(D)** (Immunohistochemical stain, x100).

**Figure 3:**
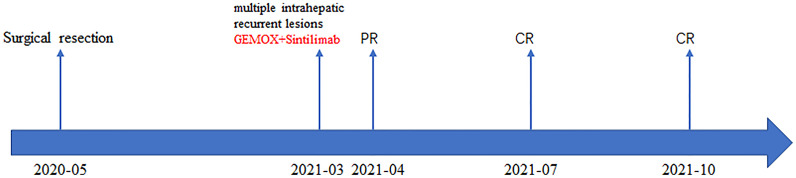
The patient’s timeline of surgery, treatment, and tumor response. PR, partial response; CR, complete response.

A 74-year-old male complained of right upper abdominal pain for several days and was admitted to the local hospital. He underwent conversion to open surgery for complete resection of gallbladder cancer and the final pathological diagnosis was GBCS. The patient did not receive any adjuvant treatment after surgery. Seven months later, the patient presented with right upper abdominal discomfort and came to our hospital for further examination. The blood routine, biochemical tests, coagulation tests, and tumor markers were within the normal ranges. The abdominal computed tomography (CT) showed multiple intrahepatic recurrent lesions in the segment of IV, V, and IV/VIII, and the largest lesion measuring 3.7×3.4 cm was located in the segment V with invasion of adjacent colonic hepatic flexure. A metastatic lesion in the hepatic hilum with a close relationship to portal vein and hepatic artery, and the main portal vein tumor thrombosis were also observed (**Figure 1A**). The Subsequent pathological consultation results of our hospital indicated a mixture of poorly differentiated adenocarcinoma and sarcomatous components, and confirmed GBCS (**Figure 2A**). The immunohistochemistry showed CDX2(+), Vimentin(+), CK19(+) (**Figure 2B**), CK(+), Ag(+), Ki67 45%, CD68(-), TTF-1(-), PSA(-), CK20(-), CK7(-), Actin(-), and PD-L1 expression of 20% the tumor cells. PD-L1 was positive in the sarcomatous components (**Figure 2C**) and negative in the carcinomatous components (**Figure 2D**). He was administered combined chemotherapy (Gemcitabine 1400 mg and Oxaliplatin 150 mg) and immunotherapy (Sintilimab 200 mg) every 3 weeks. After two courses of combination therapy, tumor shrinkage of the intrahepatic lesions, and disappearance of the lesion in the hepatic hilum and the main portal vein tumor thrombosis were observed (**Figure 1B**). All the recurrent lesions completely regressed following six courses of combination therapy (**Figure 1C**). No severe adverse event was observed during the whole course of treatment. The patient has maintained a complete response for 3 months without signs of relapse. **Figure 3** indicated the clinical course of the patient.

## Discussion

GBCS is a rare advanced BTC associated with low incidence and dismal prognosis (7). While the surgical treatment is the potentially curative option for gallbladder carcinoma, its high propensity for local invasion and early relapse may impair the surgical efficacy (8, 9). Furthermore, there is limited research on the experiences with treatment for the rare, highly malignant biliary tract neoplasm. We presented a rare case of recurrent GBCS that was successfully treated with combined GEMOX and Sintilimab. To the best of our knowledge, this is the first report of complete response in recurrent GBCS to combined chemotherapy and anti-PD-1 therapy.

Given the limited published reports regarding the GBCS, no consensus on the ideal treatment approach for the disease existed. Based on previous literatures, most of the cases received radical or palliative surgical resection, and the prognosis was dismal for the advanced disease (10). Liu et al. (11) reported 6 cases of GBCS and found the mean postoperative survival time was 2.5 months. Given the curative resection of GBCS was performed, the oncologic outcomes were still poor with a median survival time of 7.0 months (11). Of note, relatively few patients received adjuvant chemotherapy and the chemotherapy regimens were not uniforms. Pu et al. (12) reported a 59-year-old female with GBCS who was treated with oxaliplatin and 5-fluorouracil for six cycles after curative surgery, and was followed up for 6 months without recurrence. Sadamori et al. (13) reported another case of a male GBCS patient who received tegafur-uracil as postoperative adjuvant chemotherapy. The patient died 13 months after the surgery, though gemcitabine was administrated after recurrence. In another case reported by Varshney et al. (14), gemcitabine and cisplatin were administrated following the radical cholecystectomy for GBCS, and the patient achieved progression-free of disease for 6 months. Radiotherapy seemed to be a promising postoperative adjuvant therapy, although its survival benefits were based on limited evidence (15). Since targeted therapy plays an important role in the adjuvant setting, it is also included as part of adjuvant therapeutic regimes for GBCS. Shi et al. (16) reported that a female patient with recurrent sarcomatoid carcinoma of the gallbladder was refractory to chemotherapy following the palliative surgery. Therefore, anlotinib (12 mg orally every 24 hours, days 1–14, every 3 weeks) was prescribed to the patient as the alternative therapy, and partial response was observed. However, side effects led to drug withdrawal, and the patient died 5 months later. To date, there is no report on complete regression of multiple recurrent tumors following postoperative therapy. And a longer follow up was needed to determine the long-term therapeutic benefits.

In recent years, immunotherapy has shown broad prospects and revolutionized the treatment of cancer (17). Despite the remarkable progress that has been made in immunotherapy, a large number of patients fail to benefit from it (18). Thus, identifying the potent biomarkers for immunotherapy response prediction is still an unresolved issue. PD-L1 expression is often upregulated in various cancer and can predict the therapeutic response to anti-PD-1/PD-L1 blockade (19). Patients with PD-L1 overexpression in melanoma showed increased response rate to anti-PD-L1 therapy than those with PD-L1 negative melanoma (20). A phase 3 clinical trial demonstrated that pembrolizumab achieved a response rate of 44.8% and exhibited superiority over chemotherapy in terms of prognosis in patients with high PD-L1 expression (≥ 50%) advanced non-small-cell lung cancer (3). From this standpoint, the plausible explanation for the striking therapeutic effect in this case is the positive expression of PD-Ll. In this case, PD-L1 was positive in the sarcomatous components and negative in the carcinomatous components. As reported in the previous studies, PD-L1 expression was frequently found in soft tissue sarcomas, supporting the rationale for anti-PD-1 therapy in these cases (21). However, only 23% of gallbladder carcinoma expressed PD-L1 and the positive PD-L1 expression failed to show prognostic implication (22). Moreover, the previous studies reported that PD-L1 expression of advanced BTC was not associated with the treatment response of ICIs (23). Even so, these findings should be interpreted carefully, considering the small size and heterogeneity of tumors. Therefore, whether PD-L1 expression in gallbladder carcinoma has predictive value for immunotherapy response is still poorly understood and further investigations are warranted to identify the potential predictive biomarkers.

As we know, several studies have presented the supportive evidence for immunotherapy for advanced BTC (24). Pembrolizumab, an anti-PD-1 monoclonal antibody, showed sustained anti-tumor effect in 6-13% of patients with advanced BTC (25). In a multicenter phase 2 study, nivolumab monotherapy for 54 enrolled patients with refractory BTC achieved an objective response (ORR) of 22%. Among the intention-to-treat population, the median progression-free survival (PFS) and overall survival (OS) were 3.68 months and 14.24 months, respectively (6). Nevertheless, the anti-PD-1 monotherapy showed limited therapeutic effects with relatively low response rates in BTC (26, 27). Therefore, the combination strategies were introduced and achieved favorable clinical outcomes with improved efficacy and tolerable toxicity compared with the anti-PD-1 monotherapy (28). Currently, gemcitabine-based chemotherapy is the mainstay palliative treatment for unresectable BTC (29). Unfortunately, limited benefits of the adjuvant treatment were observed, necessitating novel adjuvant regimens to improve the prognosis of patients (30). It is indicated that the chemotherapy can stimulate anticancer immunity by enhancing tumor antigen presentation, activating immune effector cells, and eliminating immune suppressor cells, resulting in the synergistic anticancer effects in combination with ICIs (31, 32). In an open-label, phase II trial, Camrelizumab plus GEMOX treatment showed an ORR of 54%, a median PFS of 6.1 months and a median OS of 11.8 months in patients with advanced BTC (28). Ueno et al. evaluated the efficacy of nivolumab with or without cisplatin plus gemcitabine in unresectable or recurrent BTC. In the combination therapy group, the ORR is 36.7% with a median PFS of 4.2 months and a median OS of 15.4 months (33). While there is no discussion on the efficacy of the combined GEMOX and Sintilimab therapy for gallbladder carcinoma, the case still has important implications for future therapeutic designs.

However, this study suffers from the main limitation of a single case report with insufficient evidence to support the benefits of the treatment. The understanding of the biological behaviors of GBCS is limited owing to the rarity of the tumor. Also, the mechanisms of the current therapeutic strategies have not been clearly elucidated. Nonetheless, the present study provides a novel perspective for the treatment of GBCS.

## Conclusion

In conclusion, the present study showed a case of recurrent GBCS with positive PD-L1expression, which completely regressed following chemotherapy in combination with immunotherapy, and this rare case shed the light on the potential treatment options for unresected malignant tumors. Further studies are required to clarify the underlying mechanisms of combination therapy and improve our understanding regarding the management of the rare malignancy.

## Data Availability Statement

The raw data supporting the conclusions of this article will be made available by the authors, without undue reservation.

## Ethics Statement

Written informed consent was obtained from the individual(s) for the publication of any potentially identifiable images or data included in this article.

## Author Contributions

Q-qL, H-mL, and RZ designed and wrote the paper. H-wH, C-nY, H-mL, and CL reviewed and edited the manuscript. All authors contributed to the article and approved the submitted version.

## Funding

This work was supported by the Special Research Foundation of the National Nature Science Foundation of China (81972262, 81972255, 81772597), the Guangdong Basic and Applied Basic Research Foundation (2020A1515010117, 2018A030313645, 2016A030313840); the Fundamental Research Funds for the Central Universities (18ykpy22); Grant [2013]163 from Key Laboratory of Malignant Tumor Molecular Mechanism and Translational Medicine of Guangzhou Bureau of Science and Information Technology; Grant KLB09001 from the Key Laboratory of Malignant Tumor Gene Regulation and Target Therapy of Guangdong Higher Education Institutes; Grant from Guangdong Science and Technology Department (2015B050501004, 2017B030314026).

## Conflict of Interest

The authors declare that the research was conducted in the absence of any commercial or financial relationships that could be construed as a potential conflict of interest.

## Publisher’s Note

All claims expressed in this article are solely those of the authors and do not necessarily represent those of their affiliated organizations, or those of the publisher, the editors and the reviewers. Any product that may be evaluated in this article, or claim that may be made by its manufacturer, is not guaranteed or endorsed by the publisher.

## Abbreviations

GBCS, gallbladder carcinosarcoma; GEMOX, Gemcitabine plus Oxaliplatin; ICIs, immune checkpoint inhibitions; PD-1, programmed cell death 1; PD-L1, programmed cell death ligand 1; CTLA4, cytotoxic T-lymphocyte antigen 4; BTC, biliary tract cancer; MRI, magnetic resonance imaging; CT, computed tomography; ORR, objective response; PFS, progression-free survival; OS, overall survival.
